# Blood Biomarkers for Amyotrophic Lateral Sclerosis: Myth or Reality?

**DOI:** 10.1155/2014/525097

**Published:** 2014-06-02

**Authors:** Laura Robelin, Jose Luis Gonzalez De Aguilar

**Affiliations:** ^1^Université de Strasbourg, UMR-S 1118, Strasbourg, France; ^2^INSERM, U1118, Mécanismes Centraux et Périphériques de la Neurodégénérescence, Faculté de Médecine, 11 rue Humann, 67085 Strasbourg, France

## Abstract

Amyotrophic lateral sclerosis (ALS) is a fatal condition primarily characterized by the selective loss of upper and lower motor neurons. At present, the diagnosis and monitoring of ALS is based on clinical examination, electrophysiological findings, medical history, and exclusion of confounding disorders. There is therefore an undeniable need for molecular biomarkers that could give reliable information on the onset and progression of ALS in clinical practice and therapeutic trials. From a practical point of view, blood offers a series of advantages, including easy handling and multiple testing at a low cost, that make it an ideal source of biomarkers. In this review, we revisited the findings of many studies that investigated the presence of systemic changes at the molecular and cellular level in patients with ALS. The results of these studies reflect the diversity in the pathological mechanisms contributing to disease (e.g., excitotoxicity, oxidative stress, neuroinflammation, metabolic dysfunction, and neurodegeneration, among others) and provide relatively successful evidence of the usefulness of a wide-ranging panel of molecules as potential biomarkers. More studies, hopefully internationally coordinated, would be needed, however, to translate the application of these biomarkers into benefit for patients.

## 1. A Brief Definition of ALS

Amyotrophic lateral sclerosis (ALS), also named as motor neuron disease or Lou Gehrig's disease, is a fatal condition primarily characterized by the selective loss of upper motor neurons, which originate in the motor cortex and form the pyramidal tract, and lower motor neurons, which connect the spinal cord and brainstem to skeletal muscles. Progressive muscle weakness and atrophy, fasciculations, hyperreflexia, dysarthria, and dysphagia are common features of ALS. Of note, a significant proportion of cases presents with cognitive impairment in the form of frontotemporal lobe degeneration. Death often occurs by respiratory complications within two to five years of diagnosis. The disease typically appears between 40 and 70 years of age, and affects about two per 100,000 of people. About 90% of cases are considered as sporadic, without any documented family history. The remainder cases are most often dominantly inherited. Both forms are clinically and pathologically undistinguishable, which suggests common pathogenic mechanisms. Riluzole, which is assumed to protect motor neurons from glutamate-induced excitotoxicity, is the only accepted medication for the treatment of ALS, although its benefit is quite limited [1].

Defects in a very heterogeneous group of genes have been shown to increase the risk or to be the cause of ALS (see updated information on ALS genes at http://alsod.iop.kcl.ac.uk/) [2]. According to the relative abundance among familial (and sporadic) cases, the four most important genes causing ALS are* c9orf72* (about 40% of familial cases and 5–7% of sporadic cases), which gives rise to an expansion of an intronic hexanucleotide repeat,* sod1* (about 20% of familial cases and 2–7% of sporadic cases), which encodes Cu/Zn superoxide dismutase (SOD1),* fus* (about 5% of familial cases and less than 1% of sporadic cases), which encodes fused in sarcoma/translocated in liposarcoma (FUS/TLS), and* tardbp* (about 3% of familial cases and 1.5% of sporadic cases), which encodes TAR DNA binding protein-43 (TDP-43) [3–6]. As a result of such a genetic diversity, many mechanisms have been proposed to underlie motor neuron degeneration. Thus, excitotoxicity, oxidative stress, aberrant protein aggregation, defective axonal transport, mitochondrial dysfunction, and altered RNA metabolism have been incriminated in one way or another in the molecular and cellular pathways leading to ALS [7–12]. However, the exact nature of the selective degeneration of motor neurons still remains elusive. It is commonly accepted that the disease is the consequence of a combination of multiple pathogenic processes, which take place not only in motor neurons but also in nonneuronal neighboring cells, such as astrocytes and microglial cells and, beyond the central nervous system, skeletal myocytes and, likely, other cells in the body [13–17].

## 2. The Need for ALS Biomarkers

At present, the diagnosis of ALS is based on clinical examination, electrophysiological findings, medical history, and exclusion of confounding disorders. Although the disease is easily recognized in its full-blown presentation, during the early stages, the diagnostic process takes as long as between 13 to 18 months, since only the deterioration of symptoms and the presence of signs indicating both upper and lower motor neuron involvement can assess the existence of ALS [18]. In spite of intensive research conducted over the past 20 years, we do not currently have practical diagnostic biomarkers. This often leads to diagnostic delays before the appropriate treatment is administered. Even if riluzole is very limited in scope, it is generally acknowledged that the earlier the treatment, the more effective it is. There is also an undeniable lack of robust biomarkers that indicate the progression of ALS in clinical practice and in the context of therapeutic trials. Indeed, these are complex and long trials, because the only indicator frequently used is the average cohort survival rate. A reliable progression marker would make it possible to conduct shorter trials, on a smaller number of patients, thereby opening up the prospect of more diversified trials [19, 20].

In search of valid biomarkers to recognize ALS and pronosticate its evolution, neurophysiological approaches, such as electromyography and motor unit number estimation (MUNE), routinely play a key part in detecting lower motor neuron pathology [21, 22]. However, these methods fail to reliably monitor disease progression and hence effects of treatment. More advanced techniques, such as motor unit number index, Bayesian MUNE, and electrical impedance myography, are more accurate but still need further validation against neuropathological correlates [23–25]. As far as upper motor neuron involvement is concerned, transcranial magnetic stimulation (TMS) and its variants are useful tools to evaluate motor cortical and corticospinal dysfunction and discriminate between ALS and mimic disorders. However, at the present time, TMS techniques are mostly conducted only in the context of clinical research [26]. Besides these neurophysiological tools, neuroimaging techniques are experiencing an unprecedented development in the field of ALS research, as attested by the variety of approaches under investigation. These include radionuclide imaging techniques such as single-photon emission computed tomography and positron emission tomography, as well as conventional magnetic resonance imaging (MRI) and its derived applications: diffusion tensor imaging, functional MRI (both blood-oxygen-level dependent and resting-state), and magnetic resonance spectroscopy [27]. Over the last two decades, ALS neuroimaging has provided compelling evidence of the pathological processes occurring* in vivo* at the whole brain level, extending the notion of ALS as a multisystem disease. It has also offered promising candidate biomarkers [28]. Additional efforts must be done, however, to standardize operating procedures between clinical centers that would make these imaging techniques more widely applicable at an individual level [29].

The objective assessment of ALS and the monitoring of its progression most probably need the implementation of multipanel biomarkers, which combine neurophysiological and neuroimaging criteria with molecular parameters measured in tissues and biological fluids. At first glance, skeletal muscles represent privileged “observers” of the process of motor neuron degeneration. Thus, modifications in this tissue at the molecular level, such as alterations in gene expression or protein amounts, can appropriately monitor disease progression and the effects of disease-modifying drugs. Previous studies revealed that the neurite outgrowth inhibitor Nogo-A was expressed in muscles of ALS patients at levels that correlated with the severity of the disease [30]. Also, the analysis of the transcriptome of muscle biopsies showed that the expression of selected groups of genes distinguished between early and advanced muscle pathology in ALS patients [31]. A major limitation of these approaches relies on the fact that performing a muscle biopsy, though easily accessible, remains invasive and is not mandatory for the diagnostic examination of patients. Cerebrospinal fluid (CSF) also represents an important source of biomarkers, since it communicates directly with the brain parenchyma, and hence contains proteins and metabolites, at a concentration relatively higher than in other fluids, that can indicate the presence and extent of a neurodegenerative process. Nevertheless, from a practical point of view, blood appears to be the most suitable source for biomarker discovery; it is easy to access and handle and allows unharmful multiple testing at a low cost [20, 32]. On the other hand, it must be assumed that its composition is affected by biochemical changes in the brain and the spinal cord (or the skeletal muscles) as a result of a pathological process. Alternatively, alterations in the blood compartment could intrinsically reflect a more widespread disease sharing both central and peripheral manifestations. This minireview revisits the results of many studies that investigated systemic changes at the molecular and cellular levels in patients with ALS. When appropriate, we compared these studies to observations in the CSF, as a means to reinforce the potential of the identified blood candidate biomarkers. In essence, we performed a PubMed-based literature search on keywords related to ALS biomarkers and blood, including plasma and serum. The results are presented according to major mechanisms involved in motor neuron degeneration.

## 3. Biomarkers Related to Excitotoxicity

Excitotoxicity is a pathological process caused by the excessive stimulation of glutamatergic receptors. It occurs when the balance between release and reuptake of glutamate is disrupted, which leads to disproportionate glutamate-induced calcium influx, and subsequent neuronal toxicity and death [7]. Several blood biomarkers have been proposed in relation to this toxic pathway (Table 1). Ferrarese and coworkers [33] showed a decrease in glutamate reuptake by platelets obtained from ALS patients, and Poulopoulou and coworkers [34] found that the expression of the metabotropic glutamate receptor subtype mGLUR2, which is known to provide protection against excitotoxicity, was diminished in ALS T lymphocytes. Therefore, these findings reflected the characteristic alterations of glutamate metabolism in the central nervous system of ALS. This was confirmed by the high concentration of glutamate detected in the CSF of many patients [35]. Homocysteine has been shown to facilitate both excitotoxicity and production of reactive oxygen species. Several studies reported increased levels of homocysteine in ALS patients that correlated with the progression of the disease [36, 37]. On the other hand, it was also observed that the amount of folate, which is involved in homocysteine catabolism, was decreased, thus reinforcing the potential toxic mechanism induced by homocysteine accumulation [36].

## 4. Biomarkers Related to Oxidative Stress

Oxidative stress is the result of an imbalance between the production of reactive oxygen species, usually generated by the mitochondrial respiratory chain, and their removal from the cellular environment by antioxidant defenses. Strong evidence supports the implication of an excess of reactive oxygen species in inducing cell death in ALS [8]. Thus, numerous biomarkers indicating the presence of oxidative stress in the periphery have been proposed (Table 1). Babu and coworkers [38] reported a reduction of the activity of catalase, glucose-6-phosphate dehydrogenase, and glutathione reductase in erythrocytes of ALS patients, and this reduction was shown to be correlated with the duration of the disease. In another study, the enzymatic activity of glutathione peroxidase and SOD1 was also found to decrease in ALS erythrocytes; in addition, reduced SOD1 activity correlated with the functional status of the patients [39]. Contrasting with these findings, Tuncel and coworkers [40] reported rather high levels of SOD activity in erythrocytes of ALS patients that did not correlate with the disease status. Further discrepancies came from measures in the CSF, because both increased and decreased SOD activities were revealed [41, 42]. Along with the alteration of the activity of antioxidant enzymes, it must be added that the overall peroxyl-scavenging capacity was shown to be high in ALS patients, which was interpreted as a protective mechanism [43]. However, the amount of uric acid, which also possesses free radical scavenging activity, was decreased, and this reduction was shown to be correlated with the rate of disease progression [44]. Again, the fact that the concentration of uric acid appeared rather increased in the CSF of ALS patients contrasted with these findings [42].

Several metabolites involved in or derived from oxidative stress reactions have also been proposed as biomarkers (Table 1). Babu and coworkers [38] showed that the concentration of antioxidant glutathione was decreased in erythrocytes of ALS patients, and this reduction was correlated with the duration of the disease. On the other hand, Bogdanov and coworkers [45] found increased amounts of 8-hydroxy-2′-deoxyguanosine (8OH2′dG), which is a product of the oxidative injury to DNA. Furthermore, these authors observed that 8OH2′dG levels were higher in ALS patients with limb onset than in those with bulbar onset. The increase in the concentration of 8OH2′dG was also found in the CSF of ALS patients [46]. Similarly, the amounts of 4-hydroxy-2,3-nonenal, a product of lipid peroxidation, and prostaglandin E2, involved in free radical production, were found to be increased in the serum, as well as in the CSF [47, 48]. Furthermore, the concentration of nitric oxide, which is also involved in free radical production, was high in ALS patients, and this increase correlated with the duration of the disease [49]. Finally, it is known that alterations in the metabolism of cellular iron can induce oxidative stress. Mitchell and coworkers [50] showed increased levels of L-ferritin but decreased levels of transferrin in ALS patients, suggesting an aberrant transport and storage of iron that could lead to toxicity.

## 5. Biomarkers Related to Inflammation

ALS is characterized by an uncontrolled recruitment of microglial cells in the central nervous system and other immune cells, which contribute to motor neuron degeneration via complex and as yet misunderstood mechanisms [14]. Many of the factors involved in these inflammatory reactions can be followed in the periphery as potential biomarkers (Table 2). Therefore, the circulating levels of eosinophil-derived neurotoxin, eotaxin, granzyme A and granzyme B, high mobility group box 1 (HMGB1) autoantibody, interleukin-6, interferon-*γ*, monocyte chemoattractant protein-1 (MCP-1), tumor necrosis factor-*α* (TNF-*α*), tumor necrosis factor receptor, and wide-range C-reactive protein (wrCRP) were found to be increased in ALS patients [48–51, 57–63, 67]. In contrast, levels of granulocyte-macrophage colony stimulating factor (GM-CSF), OX40, soluble receptor for advanced glycation end products, and soluble tumor necrosis factor-related apoptosis-inducing ligand were shown to be decreased [50, 64, 66, 68]. In some cases, these changes were associated with clinical parameters. The concentrations of interferon-*γ*, MCP-1, TNF-*α*, and GM-CSF correlated with the duration of the disease [49, 50], and the levels of granzyme B, HMGB1 autoantibody, and wrCRP correlated with the degree of severity [59, 60, 63]. As observed in blood, increased levels of MCP-1 were detected in the CSF of ALS patients [58]. However, other studies reported an increase of this chemokyne only in the CSF [78].

Gene expression changes in immune cells have also been proposed as candidate biomarkers for ALS (Table 2). Thus, the expression of C-C chemokine receptor type 2 (CCR2) was decreased in monocytes obtained from ALS patients [51, 52]. In addition, it was found that patients with limb onset had less peripheral blood mononuclear cells expressing CCR2 than patients with bulbar onset [53]. Similarly, Mantovani and coworkers [52] also reported a decrease in the expression of human leukocyte antigen-DR by ALS monocytes. Reinforcing the extent of the inflammatory process, ALS patients also showed high levels of E-selectin, which is a known cytokine-induced activator of endothelial cells [56]. Finally, not only inflammatory factors but also immune cells themselves underwent quantitative modifications that could have biomarker potential for ALS. Thus, the amount of CD16+ peripheral mononuclear leukocytes was higher in patients with bulbar palsy or predominant upper motor neuron involvement than in patients with peripheral symptoms [54]. Mantovani and coworkers [52] observed less CD14+ monocytes but more CD4+ T lymphocytes in the blood of ALS patients. These authors also found less CD8+ cytotoxic T lymphocytes, although other studies rather showed opposite findings [55]. Additional studies reported an increase in the amount of natural killer T lymphocytes [55], a high neutrophil-to-lymphocyte ratio [63], and a decrease in the number of regulatory T cells [52, 55]. Moreover, the amounts of these regulatory T cells correlated with the disease progression rates [65].

## 6. Biomarkers Related to Metabolic Dysfunction

Beyond motor neuron pathology, ALS is characterized by several defects in energy homeostasis, including weight loss, hypermetabolism, and hyperlipidemia [17]. Therefore, measures of this metabolic dysfunction at the blood level can have biomarker potential (Table 3). An increase in low- to high-density lipoprotein cholesterol ratio, which is a typical index of a metabolic syndrome, was observed in ALS patients, and this increased ratio correlated with the survival rates [75]. In this respect, although the contribution of genetic variants of apolipoprotein E (APOE) to ALS is debated [79], its role in the regulation of cholesterol metabolism could influence the course of the disease. Thus, Lacomblez and coworkers [70] reported that APOE concentrations correlated with both the rate of deterioration of the patients and their survival times. As a more direct reflect of the degree of motor neuron degeneration, other studies measured the circulating concentration of the key neuronal metabolite N-acetylaspartate and found increased levels in ALS patients that correlated with the disease progression rates [76]. Similarly, the enzymatic activity of transglutaminase, viewed as an index proportional to the extent of neuronal loss, appeared, both in blood and CSF, high at the initial stages of the disease but very low at the end stages [77]. Several angiogenic factors, connected with the control of neovascularization, neurotrophicity, and metabolism, have been proposed to be involved in ALS neurodegeneration [80]. Thus, Cronin and coworkers [69] reported an increase in the levels of angiogenin that was more important in limb onset patients than in bulbar onset patients. In contrast, the amount of endoglin, another angiogenic factor, was found to be reduced [73]. Levels of insulin-like growth factor (IGF), which is known to activate anabolic pathways, were higher in ALS patients, whereas those of IGF binding protein-1 were lower, thus suggesting an impairment in the bioavailability of this trophic factor [74]. Also, levels of creatine kinase, which make ATP rapidly available to cope with energy demands, were shown to be higher in ALS patients with lumbar onset, as compared to those with bulbar onset [72]. Along with this, the concentration of ciliary neurotrophic factor (CNTF), a potent survival factor for motor neurons, was also high [71]. Taken as a whole, these changes would probably indicate leakage and/or overexpression of creatine kinase and CNTF from diseased muscles.

## 7. Biomarkers Related to Neurodegeneration

The loss of motor neurons is the primary neuropathological hallmark of ALS and, for this reason, many studies attempted to identify blood biomarkers reflecting this cell death process (Table 4). Apoptosis has been shown to play a crucial role in motor neuron cell loss in ALS [81]. Thus, Ilzecka and coworkers [82, 83] showed increased amounts of proapoptotic interleukin-1*β* converting enzyme/caspase-1 and caspase-9 in ALS patients; in addition, the increased levels of caspase-9 correlated with both the degree of severity and the duration of the disease. Similarly, a nonnegligible proportion of patients contained high levels of anti-Fas antibody, which is known to activate Fas-dependent programmed cell death [84]. Also, the concentration of cystatin C, which is a cysteine protease inhibitor involved in apoptotic neuronal cell death, was increased [85]. This study showed, however, that the amount of cystatin C in the CSF of ALS patients was rather decreased in a manner that correlated with disease progression and survival [85].

It has been postulated that the release of components of the degenerating axons into the circulation could indicate the degree of axonal pathology and hence serve as potential biomarkers for ALS (Table 4). Gaiottino and coworkers [86] reported high levels of neurofilament light chain in the serum of ALS patients, as also observed in the CSF [87]. Similarly, the concentration of phosphorylated neurofilament heavy chain (pNF-H) was increased in ALS patients [88], and this increase correlated with a faster decline [89]. In agreement with these findings, other studies also reported increased CSF levels of pNF-H in patients with ALS [90, 91]. Finally, the amount of S100-*β*, the release of which would reflect the astrogliosis accompanying the dying motor neurons, decreased during the course of the disease [92], as was also the case in the CSF [93].

## 8. Other Blood Biomarkers

Many other blood alterations have been proposed as candidate biomarkers for ALS (Table 5). The amounts of the extracellular matrix enzyme metalloproteinase-9 (MMP-9) [99] and its extracellular matrix metalloproteinase inducer (EMMPRIN) [95] were shown to be elevated in ALS patients. In addition, increased levels of EMMPRIN and MMP-2, another metalloproteinase, correlated with the severity of the disease [95, 98]. Contrasting with these findings, other studies did not observe increased levels of MMP-9 in the CSF of ALS patients, suggesting that the systemic upregulation of this metalloproteinase could indicate distal neuromuscular degeneration [99, 103]. Markers of the disruption of the extracellular milieu were also reported. Thus, the concentration of the propeptide of type I procollagen, which is an index of collagen biosynthesis, appeared low in ALS patients. In contrast, the amount of the cross-linked telopeptide of type I collagen, which is an index of its degradation, was shown to be increased in a way that correlated with the disease duration [96]. Similarly, levels of type IV collagen also correlated with the duration of the disease [102].

In accordance with studies implicating heavy metals in the pathogenesis of ALS, Fang and coworkers [97] found increased levels of lead, which was considered as a risk factor. In support of these findings, high concentrations of lead were also present in the CSF [104]. Thanks to the discovery of mutations in* tardbp* and* c9orf72*, recent studies have provided new insight into the genetic causes of ALS [3, 6]. Based on these studies, de Marco and coworkers [100] reported the accumulation of TDP-43 in the cytoplasm of circulating lymphomonocytes obtained from patients bearing* tardbp* mutations as well as from some patients without these mutations. In addition, levels of TDP-43 were increased in both blood and CSF [101, 105]. Finally, it was shown that the characteristic binding of mutant forms of C9orf72 to trimethylated residues of histones can be detected in mononuclear cells obtained from ALS patients [94].

## 9. Concluding Remarks

ALS is one of the most complex neurodegenerative diseases for which satisfactory therapeutic strategies are still lacking. Insufficient understanding of the mechanisms underlying ALS together with the absence of reliable and powerful diagnostic and prognostic biomarkers is a major cause for concern. Over the last two decades, the search for biomarkers, in particular from blood origin, has been huge. However, despite intensive research, none of the proposed biomarkers has been translated into effective tools in the clinical setting. Several obstacles must be overcome before achieving the desired results. First, the robustness of numerous studies in terms of statistical power was often not enough, because of the use of small cohorts of patients. Second, the cellular and molecular changes that were shown to be significant in ALS patients, as compared to healthy subjects, lacked specificity in many cases when compared to other neurodegenerative or neurological conditions. In this regard, potential biomarkers should also be evaluated in more appropriate control conditions that can mimic ALS, the exclusion of which causes regularly diagnostic delays during early disease stages, when patients present with only upper or lower motor neuron signs [106]. Third, different studies on the same candidate biomarker reported sometimes contradictory results. To avoid this unwanted inconsistency, both the choice of well-defined individuals and the standardization of quantification methods should be mandatory. In this respect, recent studies obtained more reassuring results when measuring pNF-H levels in the CSF to discriminate between ALS and control patients in a centralized, multicenter sample collection approach [107]. Last but not least, it should be strengthened that blood biomarkers might not always reflect the motor neuron degenerative process of ALS as those present in the CSF [108]. In fact, the blood-CSF barrier and, in particular, the blood-brain barrier could impede the crossing of diseased brain biomarkers towards the systemic compartment. Contrasting with this view, it must be also noted that the CSF is normally absorbed into the circulation on a daily basis, allowing detection of potential biomarkers in the periphery [109]. In addition, it is relatively accepted that the integrity of the blood-brain barrier is perturbed during the course of the neuropathological process of ALS [110], which would favor a leakage of molecules into the blood flow. In all, this could explain why many (but not all) of the systemic alterations affecting candidate biomarkers were associated with parallel changes in the CSF. Despite limitations and contradictory results, blood stands as an ideal source of biomarkers. A more realistic perspective arises from recent studies in the “omics” era (not treated in this review) supporting that it is likely more appropriate to identify panels of biomarkers, rather than focusing on a single gene, protein, or metabolite, in order to gain sensitivity and specificity [111–116]. Further investigations are therefore warranted.

## Figures and Tables

**Table 1:** Blood biomarkers related to excitotoxicity and oxidative stress.

Biomarker	ALS	Controls	Finding	Reference
8OH2′dG	57	27H 14ND	High plasma level Higher level in limb versus bulbar onset	[45]
Antioxidant enzyme	20	20H	Low erythrocyte activity Correlated with duration	[38]
Antioxidant status	28	20H	High serum level	[43]
Glutamate	42	40H 21ND	Low platelet uptake	[33]
Glutathione	20	20H	Low erythrocyte level Correlated with duration	[38]
GPX	88	50H	Low erythrocyte activity	[39]
HNE	85	16H 33ND	High serum level	[48]
L-Ferritin	29	36H	High plasma level	[50]
Folate	62	88H	Low plasma level	[36]
Homocysteine	62	88H	High plasma level Correlated with progression	[36]
	65	67ND	High plasma level	[37]
mGLUR2	20	20H 20ND	Low T lymphocyte expression	[34]
Nitric oxide	22	20H	High serum level Correlated with duration	[49]
Prostaglandin E2	20	20H	High serum level	[47]
Red cell SOD	25	10H	High level	[40]
SOD1	88	50H	Low erythrocyte activity Correlated with disease status	[39]
Transferrin	29	36H	Low plasma level	[50]
Uric acid	86	86H	Low serum level Correlated with ALSFRS-R	[44]

H: healthy; ND: neurological/neurodegenerative disease; 8OH2′dG: 8-hydroxy-2′-deoxyguanosine; ALSFRS-R: revised ALS functional rating scale; GPX: glutathione peroxidase; HNE: 4-hydroxy-2,3-nonenal; mGLUR2: metabotropic glutamate receptor subtype 2; SOD1: Cu/Zn superoxide dismutase.

**Table 2:** Blood biomarkers related to inflammation.

Biomarker	ALS	Controls	Finding	Reference
CCR2	42	38H 34ND	Low monocyte expression	[51]
	51	75H	Low monocyte expression	[52]
	50	40H	Low PBMC expression Less CCR2 + PBMCs in limb versus bulbar onset	[53]
CD14 + monocyte	51	75H	Low level	[52]
CD4 + T lymphocyte	51	75H	High level	[52]
CD16 + leucocyte	27	8H 9ND	High level in ALS subtypes	[54]
CD8 + T lymphocyte	51	75H	Low level	[52]
	36	35H	High level	[55]
E-selectin	25	14ND	High serum level	[56]
EDN	44	44H 82ND	High serum level	[57]
Eotaxin	20	20ND	High serum level	[58]
GM-CSF	29	36H	Low plasma level Correlated with duration	[50]
Granzyme A and B	30	30H	High serum level Granzyme B level correlated with severity	[59]
HLA-DR	51	75H	Low monocyte expression	[52]
HMGB1 autoantibody	61	40H 80ND	High serum level Correlated with severity	[60]
IL-6	20	20ND	High serum level in hypoxic patients	[61]
Interferon-*γ*	22	20H	High serum level Correlated with duration	[49]
MCP-1	85	16H 33ND	High serum level but low in later stages	[48]
	27	30ND	High serum level	[62]
	42	38H 34ND	High plasma level	[51]
	29	36H	High plasma level Correlated with duration	[50]
NK T lymphocyte	36	35H	High level	[55]
NLR	80	80H	High ratio	[63]
OX40	25	15H	Low serum level	[64]
Regulatory T cell	51	75H	Low level	[52]
	36	35H	Low level Correlated with progression	[55]
	54	33H	High level in slow progression illness	[65]
			Low level in rapid progression illness	
sRAGE	20	20H	Low serum level	[66]
TNF-*α*	20	20ND	High serum level in hypoxic patients	[61]
	22	20H	High serum level Correlated with duration	[49]
	88	40H	High plasma level	[67]
TNF receptor	88	40H	High plasma level	[67]
TRAIL	25	20H	Low serum level	[68]
wrCRP	80	80H	High level Correlated with ALSFRS-R	[63]

H: healthy; ND: neurological/neurodegenerative disease; ALSFRS-R: revised ALS functional rating scale; CCR2: C-C chemokine receptor type 2; EDN: eosinophil-derived neurotoxin; GM-CSF: granulocyte-macrophage colony stimulating factor; HLA-DR: human leukocyte antigen-DR; HMGB1: high mobility group box 1; IL: interleukin; MCP-1: monocyte chemoattractant protein-1; NK: natural killer; NLR: neutrophil-to-lymphocyte ratio; PBMC: peripheral blood mononuclear cell; sRAGE: soluble receptor for advanced glycation end products; TNF-*α*: tumor necrosis factor-*α*; TRAIL: tumor necrosis factor-related apoptosis-inducing ligand; wrCRP: wide-range C-reactive protein.

**Table 3:** Blood biomarkers related to endocrinology and metabolism.

Biomarker	ALS	Controls	Finding	Reference
Angiogenin	79	72H	High serum level High level in limb versus bulbar onset	[69]
Apolipoprotein E	403	1091ND	Plasma level correlated with progression and survival	[70]
CNTF	36	13H 30ND	High serum level	[71]
Creatine kinase	30	—	High serum level in limb versus bulbar onset	[72]
Endoglin	25	25H	Low serum level	[73]
IGF	28	28H 41ND	High serum level	[74]
IGFBP-1	28	28H 41ND	Low serum level	[74]
LDL/HDL ratio	286	369H	High plasma level Correlated with survival	[75]
N-acetylaspartate	112	51H	High serum level Correlated with progression	[76]
Transglutaminase	17	21ND	Serum activity correlated with disease status	[77]

H: healthy; ND: neurological/neurodegenerative disease; CNTF: ciliary neurotrophic factor; HDL: high-density lipoprotein; IGF: insulin-like growth factor; IGFBP: insulin-like growth factor binding protein-1; LDL: low-density lipoprotein.

**Table 4:** Blood biomarkers related to neurodegeneration.

Biomarker	ALS	Controls	Finding	Reference
Anti-Fas	52	20H 22ND	High serum level in ALS subtype	[84]
Caspase-9	30	30ND	High serum level Correlated with severity and duration	[83]
Cystatin C	44	35H 25ND	High plasma level	[85]
ICE/Caspase-1	25	15H	High serum level	[82]
NFL	46	67H 97ND	High serum level	[86]
pNF-H	62	—	Plasma and serum level correlated with ALSFRS-R decline	[89]
	71	40H 52ND	High plasma level	[88]
S100-*β*	41	32H	Serum level correlated with progression	[92]

H: healthy; ND: neurological/neurodegenerative disease; ALSFRS-R: revised ALS functional rating scale; ICE: interleukin-1*β* converting enzyme; NFL: neurofilament light chain; pNF-H: phosphorylated neurofilament heavy chain.

**Table 5:** Other blood biomarkers.

Biomarker	ALS	Controls	Finding	Reference
C9orf72	2	2H	Mononuclear cell binding of mutant c9orf72 to tri-CH_3_ histone residues	[94]
EMMPRIN	50	50H	High serum level Correlated with severity	[95]
ICTP	21	16ND	High serum level Correlated with duration	[96]
Lead	184	194H	High level Correlated with risk of ALS	[97]
MMP-2	30	15H	Correlated with severity	[98]
MMP-9	14	20H 45ND	High serum level	[99]
PICP	21	16ND	Low serum level	[96]
TDP-43	16	13H	Cytoplasmic lymphomonocyte location in ALS subtype	[100]
	219	100H	High plasma level	[101]
Type IV collagen	30	30H 14ND	Correlated with duration	[102]

H: healthy; ND: neurological/neurodegenerative disease; EMMPRIN: extracellular matrix metalloproteinase inducer; ICTP: cross-linked telopeptide of type I collagen; MMP: matrix metalloproteinase; PICP: propeptide of type I procollagen; TDP-43: TAR DNA binding protein-43.
